# Recurrent cutaneous basal cell carcinoma after surgical excision: A retrospective clinicopathological study

**DOI:** 10.1016/j.amsu.2022.103877

**Published:** 2022-05-26

**Authors:** Abdulkarim Hasan, Ahmed Rabie, Mohammed Elhussiny, Mohamed Nasr, Mohamed I. Kamel, Ahmed Hegab, Abdelrahman S. El-Kady, Mahmoud E. Nagaty, Abdelhafez Seleem, Mohammed Abbas, Abd Al-Kareem Elias, Gamal G. Shemy, Ahmed Abu_Elsoud, Asmaa A. Dahy, Ayman Abdulmohaymen, Ahmed Youssef, Ayman Abdelmaksoud

**Affiliations:** a Pathology Department, Faculty of Medicine, Al-Azhar University, Cairo, Egypt; b Pathology Department, Damietta Faculty of Medicine, Al-Azhar University, Damietta, Egypt; c Histology Department, Faculty of Medicine, Al-Azhar University, Cairo, Egypt; d Dermatology, Venerology and Andrology Department, Faculty of Medicine, Al-Azhar University, Cairo, Egypt; e General Surgery Department, Faculty of Medicine, Al-Azhar University, Cairo, Egypt; f General Surgery Department, Faculty of Medicine, Al-Azhar University, Assiut Branch, Egypt; g Plastic Surgery Department, Faculty of Medicine, Al-Azhar University, Assiut Branch, Egypt; h Plastic Surgery Department, Faculty of Medicine for Girls, Al-Azhar University, Cairo, Egypt; i Surgical Oncology Department, Faculty of Medicine, Al-Azhar University, Cairo, Egypt; j Mansoura Dermatology, Venerology and Leprology Hospital, Mansoura, Egypt; k Department of Dermatology, University of Studies Guglielmo Marconi, Rome, Italy

**Keywords:** Basal cell carcinoma, Excision, Recurrence, Skin cancer, Surgical margin

## Abstract

**Background:**

Recurrence of basal cell carcinoma (BCC) after complete surgical excision is rarely reported. Risk factors for this negative outcome are not well-studied. We present the clinical and histological features of recurrent BCCs in our institution.

**Methods:**

All patients between January 2016 to December 2020 whose primary BCCs were excised with free surgical margins according to the histopathology report, and represented later with local recurrence were included. The medical files were retrieved to record patient's age, sex, sun exposure, tumor site, size, clinical diagnosis, histopathology variant of primary lesion, least free margin distance of the original lesion, and recurrence time.

**Results:**

Eighteen patients (11 males and 7 females ranged between 50 and 75 years old) fulfilled the inclusion criteria; all of their lesions were located in head and neck regions. The mean recurrence time was 31.2 months (11–86) and the histological variant was the same of primary in 17/18 patients. Primary tumors showed nodular subtype in 77.8% of patients and 55.6% of the primary tumors were less than 15 mm in diameter. Sun exposure history was given by 77.8% of patients while the rest of patients had non-significant exposure. All recurrent excised lesions were of free margin less than 4 mm.

**Conclusion:**

We found that the primary tumors of all studied recurrent BCCs were excised with surgical margins less than 4 mm. We recommend follow up for all excised BCCs either those of low or high risk histological variants. Tumor size does not appear a considerable risk factor for local recurrence.

## Highlights

• We report recurrent BCCs in 18 patients, all were located in head and neck region.
• Sun exposure history was given by 77.8% of patients while the rest of patients gave a negative history of sun exposure.
• Nodular histological subtype was seen in 77.8% of patients.
• All primary tumors were excised by general or plastic surgeons with a surgical margin less than 4 mm.

## 1 Introduction

Basal cell carcinoma (BCC) is the most frequent cutaneous cancer that accounts for 70–80% of all skin malignancies with variable clinical presentations, histological features, and biological behavior [1,2]. Low risk BCC mostly affects the trunk, extremities, scalp, forehead, cheeks, and neck. Histological subtypes of low risk BCCs includes superficial and nodular subtype. Low risk BCCs lack perineural invasion (PNI), while high risk tumors are larger size, mostly affect the trunk and extremities, and middle face. Histologically, high risk BCCs are poorly defined with PNI. High risk BCCs tend to be recurrent [3]. Surgical excision with safety margin is the standard therapeutic line of cutaneous BCC with potential lower rates of recurrence and metastasis post-operatively [4]. Five-year local recurrence rates for standard surgical excision of BCCs varied from 2.3% to 10.1% [5]. A four mm safety margin was recommended for low-risk lesions [3].

Recurrent BCCs may present with localized erythema, induration or ulceration at the site of surgical excision of the primary lesion [1]. A limited data in the literature are available on demographic, clinical, and histopathological predictors of BCCs recurrence [6].

This study we aim to identify the clinical and pathological criteria of recurrent BCCs, including topographical localization, clinical presentations, size of the lesions, histological variants and surgical margin's status of the primary excised lesion, over a 5 year-period (between January 2016 to December 2020) at Al-Azhar University Hospitals in Egypt.

## 2 Methods

This is a retrospective clinicopathological study performed at Al-Azhar hospitals after getting bioethical committee approval number IRB00012367-22-02-004. We reviewed all the patients between January 2016 to December 2020 who had surgically-excised primary BCCs with safety margin, and presented to dermatology or different surgical departments with local recurrence of such excised lesions. We obtained patients' data from the medical records and surgical pathology database. Inclusion criteria include all the patients who had pathologically-confirmed, surgically-excised BCCs with safety margins, and those with clinic-pathological diagnosis of recurrent BCCs. Exclusion criteria include non-excised primary lesions, involved surgical margins of the primary, patients underwent Mohs' micrographic surgery or those with incomplete clinic-pathologic diagnosis of the recurrent lesions. All the data regarding patient's age, sex, occupation, anatomical site of the lesion, clinical diagnosis, tumor size, histopathology variant of primary lesion, least free margin distance of the primary lesion, period between primary excision and the recurrence were recorded and retrieved. Tumors with multiple subtypes or variants were analyzed based on the highest risk variant; infiltrative/sclerosing/morphea form (referred to as “infiltrative” only) superseded micronodular, which superseded nodular. Nodular and pigmented referred as “nodular”. STROCSS criteria was followed in this work [7].

Statistics: Data was managed by the Excel program (the Microsoft Corporation, Redmond, USA). Results were recorded as the mean ± SD for age and tumor size. Frequencies and percentages were computed for the descriptive variables. A P-value of less than (0.05) was considered statistically significant. The Interrater reliability between clinical diagnosis and final pathological diagnosis was assessed statistically by the kappa test.

## 3 Results

A total of 18 patients who underwent surgical excision with safety margins for cutaneous BCC presented with local recurrence of the excised lesions in a ranged timeframe from 11 months to 84 months post-operative. The average age of those patients was 63.7 + 7.9 years (ranged from 50 to 75 years), including 11 males and 7 females. 14/18 experienced excessive sun-exposure during this period, whereas 4 patients (housewives) had no similar history (Table 1).

**Table 1:** Clinico-histopathological characteristics of the tumors.

Characteristic	N (18)	%
**Age (Years)**		
Mean ± SD	63.7 ± 7.9	
Range	50–75	
**Sex**		
Male	11	61.1
Female	7	38.9
**Regarding sun exposure**		
Sun exposed patients	14	77.8
Not sun exposed patients	4	22.2
**Sites of the lesions**		
Nose	8	44.4
Scalp	3	16.7
Ear	2	11.1
Forehead	2	11.1
Medial canthus	2	11.1
Cheek	1	5.6
**Clinical diagnoses**		
BCC	16	88.9
Other than BCC	2	11.1
**Tumor size**		
Mean ± SD	16.5 ± 9.3 mm	
Range	5–40 mm	
**Tumors classification according to size**		
<15 mm (small size)	10	55.6
≥15 mm (large size)	8	44.4
**Tumors classification according to histopathological diagnoses**		
low risk (Nodular subtype)	14	77.8
High risk (Infiltrative subtype)	4	22.2

**Fig. 1:**
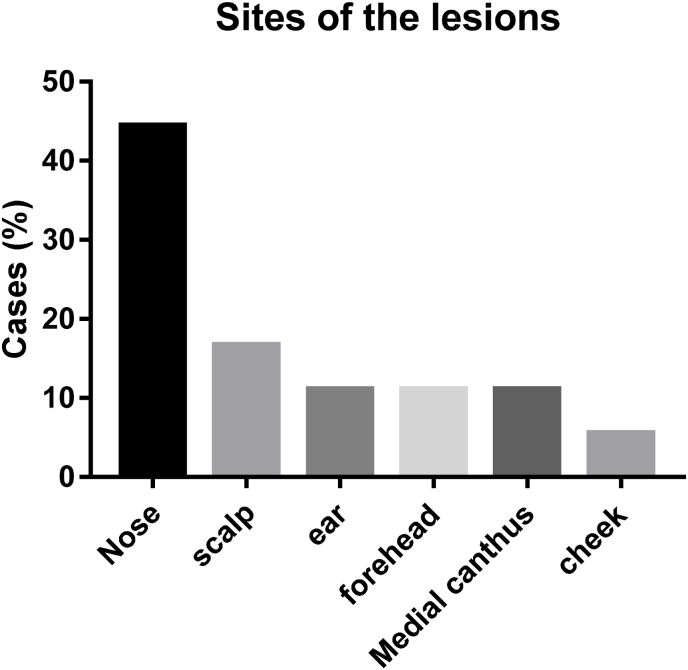
A bar chart showing the percent of cases related to the sites of the tumors.

All the lesions located on the head and neck region in a descending frequency as the following; 8 on the nose, 3 on the scalp and 2 lesions on multiple sites (i.e. forehead, ear and eye) and one lesion on the cheek (Fig. 1, Table 1).

**Fig. 2:**
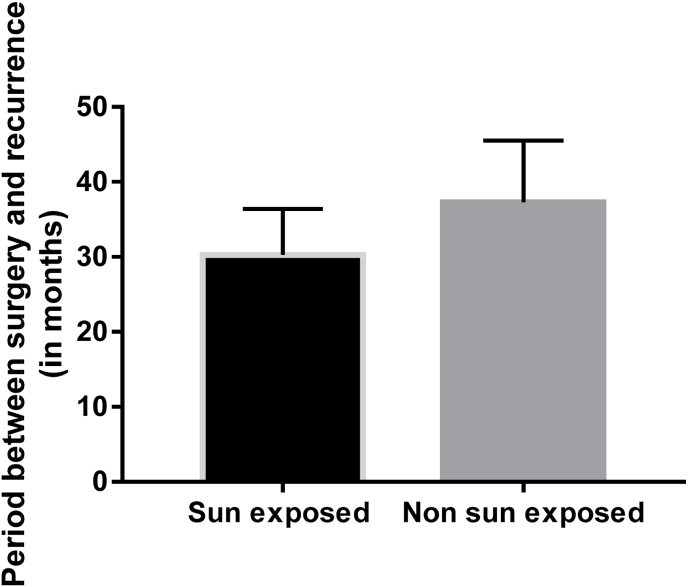
A bar chart showing the comparison between BCC in sun exposed and non-sun exposed patients in relation to the period between surgery and recurrence. N: Sun exposed = 14, non-sun exposed = 4; Unpaired *t*-test. (Data are shown as means + SEM).

To examine the relation between cutaneous BCCs local recurrence and sun exposure, we compared between sun-exposed and non-sun exposed patients in relation to the time period between surgical excision and BCCs recurrence. We did not find a significant difference between sun-exposed and non-sun exposed patients regarding the timing of post-operative recurrence (Fig. 2; unpaired *t*-test, t = 0.5635, p > .05).

**Fig. 3:**
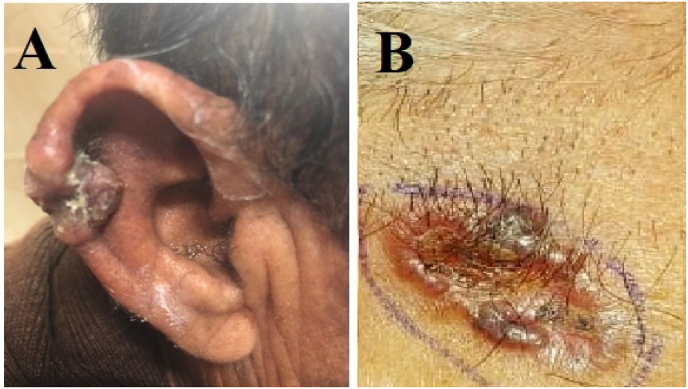
Clinical picture of two BCCs on the head and neck region; right ear (A) and scalp (B).

Two patients were referred for a clinical suspicion of melanocytic lesion and nodule of uncertain etiology, respectively. The rest of the patients (16/18) were referred with a provisional diagnosis of BCCs (Fig. 3). The clinical–histological agreement percentage for detection diagnosis confirmation was 88.8%. The applied Interrater reliability, Cohen's kappa coefficient (κ) showed substantial agreement. It was suggested by Dr Cohen that Kappa results be interpreted as follows: values ≤ 0 indicating no agreement and 0.01–0.20 as none to slight agreement, 0.21–0.40 as a fair, 0.41–0.60 as a moderate, 0.61–0.80 as a substantial, and 0.81–1.00 as almost perfect agreement [8].

The tumor size of the primary lesions (according to the gross pathology measurements) ranged from 5 mm up to 40 mm with mean size 16.5 + 9.3. We categorized the studied tumors into 2 groups; Small size (less than 15 mm) and large size (more than 15 mm) (Table 1).

**Fig. 4:**
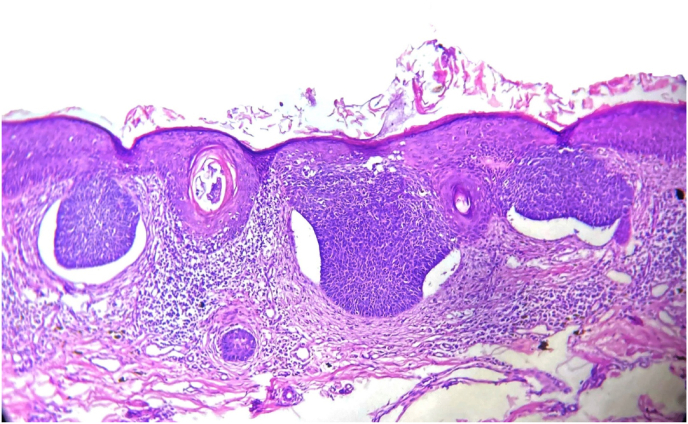
A histopathology picture of a nodular BCC showing slightly acanthotic epidermis with underlying basaloid malignant cells in nodular pattern with peripheral palisading and retraction artifact (H&E stain, 100x).

The histopathological diagnosis were categorized into low risk, including 14/18 of the primary lesions (all were nodular subtype, see Fig. 4), and high risk subtypes which were the rest of tumors (4/18) (all were infiltrative) (Table 1).

The recurrent lesions showed the same features of the primary subtype in 17/18 patients, and only one patient who had shown a nodular subtype in the primary lesion, but recurred as an infiltrative subtype.

Least surgical margin for the primary lesions where as follows; 14 primary tumors were excised with least free margin 1 mm, 3 patients with 2 mm and one patient with least free margin 3 mm (Table 2). The timeframe between the first operation and the appearance of recurrence (recurrence time) ranged from 11 month up to 86 months, mean period 31.2 + 19 (Table 2).

**Table 2:** Surgical margin status for the primary lesion of the tumors and time of recurrence after surgical operation.

**Margin status**	
Mean ± SD	1.3 ± 0.57 mm
Range	1–3 mm
**Time between surgical operation and recurrence**	
Mean ± SD	31.2 ± 19 months
Range	11–86 months

## 4 Discussion

Cutaneous BCC is a malignant skin tumor derived from epidermal cells, mostly from hair follicle stem cells [9,10]. Others considered BCC stems from the infundibulum and interfollicular epidermis [11].

Diagnosis of BCC depends on clinical suspicion and investigating tools, mainly histopathological examination. Dermoscopic examination increases diagnostic sensitivity to 91% and specificity to 95%. Small nodular subtype on the head/neck and trunk, multiple BCCs of naevoid basal cell carcinoma syndrome and superficial subtypes on the trunk or extremities, can solely diagnosed based on combined clinical and dermoscopic examinations [12].

However, histopathologic examination of the excised tissue is the main diagnostic tool for BCCs lesions [13]. Histopathology would verify low-risk the high-risk subtypes of BCCs. Histopathology would give an overview on the excised margins, verify the histological type (e.g. micronodular, morpheaform/sclerosing, infiltrative, or basosquamous), depth of invasion (e.g. invasion beyond the reticular dermis is a feature of aggressive tumor), lymphovascular invasion and perineural involvement [14,15]. Histopathologic examination is not only to establish an accurate diagnosis of BCCs, but also to provide a predictive information on the tumor behavior and risk of possible recurrence [14].

Etiopathogenesis of recurrent BCC is multifactorial. Prior studies on BCCs have focused on the risk factors which increase the rate of tumor local recurrence. Several clinical and histological features have been associated with possibility of recurrence, such as, larger tumor size, lesions of the head/neck, poorly defined borders, incompletely excised lesions, perineural invasion [16,17].

In one study, a 15% five-year recurrence rate for surgically excised BCCs was for lesions larger than 15 mm in diameter. The authors considered increased tumor size a significant predictor for recurrence when adjusted for lesions on the ears, eyes, scalp, nose, or face [17] Large BCCs had a 9% risk of recurrence, compared to 0.8% risk for small BCCs [18].

Our results revealed that 56.25% of recurrent BCCs were larger than 15 mm in diameter vs 43.75% less than 15 mm in diameter. That might means risk of recurrence is not limited to larger lesions only, but also the small sized lesions. We did not record recurrent lesions in other location outside the head/neck region, which is consistent with Vornicescu et al. study in 2021 [19]. Vornicescu et al. reported 8 recurrent BCCs; all of them were head and neck lesions. Morgan et al., in 2019 [18] recorded 4 out of 22 of recurrent BCCs lesions located outside the head and neck. Boulinguez et al., in 2004 recorded one out of 33 recurrent lesions were on the back [20].

Ultraviolet radiation has been reported as one of the most common risk factors of BCC development [21] hence, head and neck are the most common anatomical sites of BCCs development. Sun exposure was recorded in 87.8 (14/18) of our patients, whereas 22.2% (4/14) of the patients who had recurrence after surgical excision with safety margin had negative history of sun exposure. That means underlying triggering factors for primary and recurrent BCCs other than ultraviolet light exposure. Several studies on BCC reported higher number of females who had no history of excessive sun exposure [22]. However, male patients were more predominant in our study, about 60% of all patients, which comes compatible with previous studies for recurrent BCCs [6,18].

Immunosuppression may play a role in BCCs recurrence in our cohort. All the sun protected patients were older than 65 years with immune senescence. Immunosuppression has been considered one of the risk factors of non-melanoma skin cancers [23]. Others did not find a significant high risk for recurrence among patients with immunosuppression [24]. All the patients in this study were over 50 years old (up to 75 years) with mean age of 63.7 which is a slightly different from patients in France according to Boulinguez et al. [20] who noted a mean age of 69 years (range 41–91) among those recurrent BCCs. Morgan et al., in 2019 [18] found the same result (mean age 64) and recorded two immunosuppressed patients out of studied 22 recurrent BCC patients. Actually, male gender older than 60 years old age posed a significant increased risk of BCC recurrence [25]. Others noted a non-significant difference between recurrent and non-recurrent BCCs based on the age and gender of the studied patients [26].

Time lapsed from surgical excision of the primary lesion to recurrence of the lesion in our study ranged from 11 to 86 months (mean 31.2 ± 19 months). The shortest period was detected in a patient who experienced surgical excision with least free margin of 3 mm. That means recurrence could be expected within one year of surgical excision. The average time of recurrence recorded by Knani et al. [27] in 2014 was 73.8 months. Bartoš et al. [28] recorded a mean interval between the primary and subsequent secondary lesion of 31.2 months (ranged from 4 to 105 months), with the majority of lesions appeared within 3 years. It was 2 years according to a recent study [19]. This variation between the studies may be related to the ethnicity of the studied cohort. Morphologically, recurrent tumors in this study were 14/18 (77.8%) nodular subtype of BCC which is considered a low risk histological variant and 22.2% infiltrative (high risk) subtype, and subsequent morphology for the recurrent lesions was consistent with their original histomorphology in 17/18 (94.4%) patients and only one patient presented by a more aggressive histological subtype in the recurrence (from nodular to infiltrative). Nodular subtype carcinomas in our study was predominant, which may be considered unusual finding and differs from most of the previous peer studies; Vornicescu et al., in 2021 [19] found 50% nodular and 50% infiltrative, Morgan et al. [18] revealed 2 patients of nodular versus 17 patients of infiltrative subtype and classified recurrent BCCs in their study into aggressive variants and Indolent-growth variants and latter percent was 45%.

Total surgical excision with safety margins is still considered the ‘gold standard’, and most BCCs are easily treated with complete surgical excision giving low rates of recurrence and metastasis [4,28]. No significant difference in recurrence of BCC between surgically excised group and Mohs' micrographic surgery group for most patients studied by Mosterd et al. [29], but latter is preferred over surgical excision for facial recurrent BCCs. The NCCN recommends a 4 mm safety margin for low-risk lesions. We noted all recurrent BCCs in this study were previously excised with less than 4 mm safety margin.

Limitations of this study include the number of the studied cases which is only 18 patients in addition to lack of information about the other potential risk factors such as polyaromatic hydrocarbons and arsenicals which are reported by Varan et al. [30]. The reported rates of BCC recurrence vary widely in the range of 0.5–38% in different studies [28] but one of our study limitations is the inability to calculate the recurrence rate in our institution. Some authors performed some useful mmunohistochemical staining for BCCs [31] but here we depend only on the ordinary stains with carful microscopic examination by expert histologists and anatomical pathologists.

## 5 Conclusion

The NCCN recommends a 4 mm safety margin for low-risk lesions, here we found that the primary tumors of all the studied recurrent BCCs were excised with surgical margins less than 4 mm. Follow up is recommended for all excised BCCs either those of low or high risk histological variants. Size of the primary lesion does not appear a considerable risk factor for local recurrence.

## Provenance and peer review

Not commissioned, externally peer-reviewed.

## Ethical approval

Ethical approval was obtained from Damietta Faculty of medicine, Al-Azhar University under ID number: IRB00012367-22-02-004

## Source of funding

This study did not receive any funding from governmental or private organizations.

## Author contribution

Study concept or design: AH, AR, ME, MN, MIK, AHeg, ASE, AS, MA, MEN, AE, AA, AAbu, Data collection: AH, AR, ME, MN, MIK, AHeg, ASE, AS, MEN, AE, GGS, AA, AAD, AAbdu, Data interpretation: AH, AR, ME, GGS, AA, AAD, AAbdu, AY, A Abdel Literature review: AH, AR, ME, MN, MIK, AHeg, ASE, MA, MEN, AE, A Abdel Data analysis: AH. ME. Drafting of the paper: ALL. Editing of the paper: ALL. Manuscript revision: ALL.

## Registry number

Identifier: NCT05267691.

## Guarantor

Dr. Abdulkarim Hasan.

## Consent

Provided.

## Declaration of competing interest

The authors declare no conflict of interest.
